# Assessing the Acceptability of a Co-Produced Long COVID Intervention in an Underserved Community in the UK

**DOI:** 10.3390/ijerph182413191

**Published:** 2021-12-14

**Authors:** Sally Fowler-Davis, Rachel Young, Tom Maden-Wilkinson, Waqas Hameed, Elizabeth Dracas, Eleanor Hurrell, Romila Bahl, Elisabeth Kilcourse, Rebecca Robinson, Robert Copeland

**Affiliations:** 1 Advanced Wellbeing Research Centre, Sheffield Hallam University, Sheffield S9 3TU, UK; S.Fowler-Davis@shu.ac.uk (S.F.-D.); tm7800@my.shu.ac.uk (T.M.-W.); R.J.Copeland@shu.ac.uk (R.C.); 2 Community Wellbeing Service (Specific Identity Withheld); w.hameed@darnallwellbeing.org.uk; 3 Sheffield Health and Social Care NHS Foundation Trust, Sheffield Teaching Hospitals NHS Foundation Trust, Sheffield S5 7AU, UK; e.draca@nhs.net; 4 Royal Hallamshire Hospital, Sheffield Teaching Hospitals NHS Foundation Trust, Sheffield, S10 2JF, UK; ellie.hurrell@nhs.net; 5 Faculty of Sport and Exercise Medicine, Virtual Institution, Edinburgh EH8 9DR, UK; romila.bahl@nhs.net (R.B.); elisabeth.kilcourse@nhs.net (E.K.); Rebecca.Robinson@eis2win.co.uk (R.R.)

**Keywords:** Long COVID, rehabilitation, virtual methods, multi-disciplinary team

## Abstract

Background: The COVID-19 pandemic has disproportionately affected people from more deprived communities. The experience of Long COVID is similarly distributed but very few investigations have concentrated on the needs of this population. The aim of this project was to co-produce an acceptable intervention for people with Long COVID living in communities recognised as more deprived. Methods: The intervention was based on a multi-disciplinary team using approaches from sport and exercise medicine and functional rehabilitation. The co-production process was undertaken with a stakeholder advisory group and patient public involvement representation. This study identified participants by postcode and the indices of multiple deprivation (IMD); recruitment and engagement were supported by an existing health and wellbeing service. A virtual ‘clinic’ was offered with a team of professional practitioners who met participants three times each; to directly consider their needs and offer structured advice. The acceptability of the intervention was based on the individual’s participation and their completion of the intervention. Results: Ten participants were recruited with eight completing the intervention. The partnership with an existing community health and wellbeing service was deemed to be an important way of reaching participants. Two men and six women ages ranging from 38 to 73 were involved and their needs were commonly associated with fatigue, anxiety and depression with overall de-conditioning. None reported serious hardship associated with the pandemic although most were in self-employment/part-time employment or were not working due to retirement or ill-health. Two older participants lived alone, and others were single parents and had considerable challenges associated with managing a household alongside their Long COVID difficulties. Conclusions: This paper presents the needs and perspectives of eight individuals involved in the process and discusses the needs and preferences of the group in relation to their support for self- managed recovery from Long COVID.

## 1. Introduction

COVID-19 is the disease associated with the SARS-CoV-2 virus which has led to substantial morbidity and mortality globally [1] with disproportionate affect upon deprived communities and people of diverse ethnicity [2]. Material deprivation has been identified as a key predictor of COVID-19 outcome and interventions to target inequality need to be prioritised [3]. Challenges associated with housing, type of employment and prevalence of co-morbidities have been associated with higher infection rates and mortality amongst deprived communities [4]. The long-term sequelae of COVID-19 including persistent symptoms and impact upon mental health are strongly influenced by social determinants such as poverty, social disadvantage and structural inequality [5,6].

Over the past decade, the role of patients and the public in research and service planning has developed with a range of co-production methods used to involve and purposefully engage those who will benefit [7]. When patients contribute to the research process and engage in research-related activities, they move from a passive role as subjects, to research participants who can comment fully on the acceptability of the treatments being planned or so-called complex interventions [8]. A key question in evaluating a complex intervention is about practical effectiveness and whether the intervention works in everyday practice [9].

The aim of this project was to co-produce an acceptable intervention for people with Long COVID, living in communities recognised as more deprived. A case report approach was adopted to evaluate the intervention, taking account of individual circumstances to capture a participant centred perspective [7,8]. Long COVID was the term adopted by patients based on self-reported persistent symptoms following COVID-19 infection [10] that include (but are not limited to) fatigue, breathing difficulties and an associated anxiety about an often-dramatic decline in wellbeing, physical and psychological function. The relapsing/remitting nature of Long COVID symptoms are an important consideration for those experiencing difficulties [11] and care providers, with persistent symptoms recognised as being chronic beyond 12 weeks post infection. Recognising the disproportional effect of COVID-19 in more deprived communities [12] this project sought to include acceptable methods for recruiting and engaging people in an underserved area of social and economic deprivation, so that learning can be shared widely.

The acceptability of a virtual Long COVID intervention, includes targeting participation with a more deprived or underserved community, and reflects the organisational learning needs of the current and ongoing development of new National Health Service (NHS) services in England [13]. There is historical and contemporary evidence of some communities who have more limited access to care and health services [14] with accumulated or aggregated disadvantage rather than ‘simply’ material deprivation exasperating health inequalities and the implementation design needs to reflect this knowledge. Some people of diverse ethnicity demonstrate greater levels of pre-existing chronic health conditions and higher levels of chronic illness and this may be a product of socioeconomic disadvantage and racial discrimination [15] and so there is a likelihood that the incidence of Long COVID is higher in ethnically diverse communities. However, there are limited examples of co-production of health interventions with deprived communities [16] that might achieve improvements in service uptake and subsequent better health outcomes.

Population health perspectives are an increasingly important framework in healthcare [17] with an aim to reduce the cost of long-term care for disability and increase the impact of health interventions. In addition, the research team were interested in an opportunity to demonstrate how sport and exercise medicine, in the context of an interdisciplinary care team, was able to extend their traditional focus (on athletes) and engage in a population health program. The co-production of the virtual, multidisciplinary Long COVID clinics undertaken during a lockdown period of early 2021 and is reported here as a case report reflecting the new illness of Long COVID and the sPPI that supported and enabled the engagement within a particular context including people with diverse ethnicity in an area of deprivation. The development of the intervention is intended to support learning for practitioners and planners across the health and care systems and include those who may contribute to the wider social recuperation from Long COVID, including those from sport, leisure services and community services.

## 2. Materials and Methods

Qualitative research focuses on the study of “things in their natural settings, attempting to make sense of, or to interpret, phenomena in terms of the meanings people bring to them” [18]. Co-production is necessarily qualitative in nature but extends the ambition of the inquiry to explore issues of power and control [19] in society, communities, and particular practices. It seeks to better understand and ultimately level up the current systems in which healthcare operates. Co-production facilitates participation as a core principle. The co-production process engages participants in self-determined action, and participation involves the negotiation and transformation of structural and institutional control [20].

The invitation to participate in the research was intentionally designed to enable people with knowledge and experience of long COVID to express preferences; to select best-fit goals and challenge medical assumptions to produce a more person-centred intervention. This was operationalised as a participatory clinical research process, a “systematic inquiry, with the collaboration of those affected by the issue being studied, for purposes of education and taking action or effecting change” [21]. There were two phases to the co-production, the first based on informed opinion and patient and public involvement (PPI) and the second phase, a virtual clinic that enabled participant ‘recruits’ to respond to questionnaires, give feedback on the perception of the challenge of on-line/virtual conversations and share their response to the interventions. The combination of the two phases was used as feedback and data; enabling the researchers to evaluate the acceptability of the offer. This method was used to ensure that those from a more deprived community were as fully participative as possible and enabled to voice their preferences and engage in the intervention planning. A case report approach was adopted to the analysis of data generated during phase two to facilitate an individualised perspective on each participant’s experience of the co-produced intervention.

### 2.1. Development of the Intervention

The intervention was co-produced with recruitment over a four-month period from November 2020 and was funded by Sport England as part of National Lottery funding via Sport England. The study ethics was approved through Sheffield Hallam University (ER29953169) based on three independent reviewers’ scrutiny of the purpose and participant involvement. The initial phase of the project entailed team formation, in particular the contractual arrangements with an existing local organisation for the Health and Wellbeing (H&W) link worker. The study design was to achieve a rehabilitation intervention and this contrasted with the usually medical literature and so the application of expertise to emergent new knowledge of Long COVID [22]. Detailed clinical governance arrangements were drawn up and approved by senior leadership groups within the university.

Phase one aimed to co-produce a tailored intervention through consultation with expert service providers and service users. A stakeholder advisory group was formed which included representatives of practitioners, and members of the public with experience and interest in symptoms associated with Long COVID. In addition, volunteers from an existing Patient Public Involvement (PPI) group attached to the research centre had local knowledge of the area of planned recruitment and contributed to the co-production process. The group met formally on two occasions and advice was used to co-design a complex intervention, based on rehabilitation principles but using evidence of likely preferences and take up from the local community. In consultation with the Primary Care Network (PCN), follow up letters post intervention were circulated to the General Practitioners (GP) of participants to summarise their engagement with the study and reported symptoms. The co-produced protocol involved a guided ‘conversation’ held over three remote sessions scheduled according to each participant’s preferred availability.

Phase two aimed to implement and evaluate the co-produced protocol using a case report approach to data analysis. A clinical academic delivery that could be achieved over a four-month period was implemented; recruiting participants who lived in an underserved community. The Health and Wellbeing (H&W) worker was trained in the process of informed consent and a standard participant information sheet was used to invite participants. This was a face-to-face process adopting usual access that was typical of the H&W role in the community. The diversity of those engaged was important, needing to reflect the age and gender demographic as well as diversity in relation to ethnicity and family households. The employment and household status were evident as a key factor in recuperation and rehabilitation with specific unpaid gender roles and recognition that consideration should be given to the strain factors associated with unpaid family work when designing and organising interventions to promote access to medical rehabilitation services [23].

Participant recruitment was planned to identify and engage people against an inclusion and exclusion criteria that supported purposive recruitment. In this case, the participants were all identified by geographical location. All participants lived in a single ward that is recognised as within the 10% most deprived nationally and is the third most deprived neighbourhood in the city. Each participant’s postcode was mapped to the IMD 2015 [24] and the IMD rank and decile between one and five. The Indices of Multiple Deprivation (IMD) allows for a classification by area and so identifies relative advantage and disadvantage rather than by individual status. Guidance on the development and evaluation of complex interventions [8] includes several interacting components that are sensitive to local context.

Outcome measurement methods were selected to capture the self-reported functional and demographic status of participants at entry to the project alongside self-reported quality of life and symptoms. A selection of validated tools was transferred into an on-line format using Qualtrics and were call ‘modules’ for the participant to complete from a link on their mobile phone or computer. Module 1 was formal consent and demographic data, Module 2–4 the on-line (Qualitrics) versions of the tools that formed a ‘minimum data set’ on each participant. Prior to session one, participants were required to complete an on-line self-report questionnaire which comprised the Euro-Quality of Life-5D (QOL-5D) [25] an internationally validated quality of life assessment with five items and an analogue score about the overall burden of disease between one and 100, the Fatigue Severity Scale (FSS) [26], a nine-item scale which measures the severity of fatigue and its effect on a person’s activities and lifestyle in patients with a variety of disorders. The Post COVID Functional Status Scale (PCFSS) [27] was also used, to measure the full spectrum of functional outcomes following COVID-19. In addition, self-reported age, employment status and household composition, GP name, postcode information were included in the minimum data set.

The protocol incorporated advice regarding gradually increasing engagement in physical activity tailored to each participant’s current level of activity, reported priorities and symptoms. This could include recommendation for a short duration low intensity activity such as a short walk in the local park, with advice on mirrored rest and pacing. The protocol included guidance on engagement in activity within individual levels of exercise tolerance incorporated with pacing of activity routines. Monitoring of symptoms was emphasised and, where appropriate, daily wellness scoring was introduced. On completion of the third session, participants were required to repeat the self-report questionnaires plus the patient experience CARE Measure [28]. The Consultation and Relational Empathy (CARE) Measure is a person-centred process measure that was developed and researched at the Departments of General Practice in Glasgow University and Edinburgh University. The CARE Measure has ten questions and is a participant-completed questionnaire relating to the relational elements of the intervention.

The design of the intervention was undertaken with advice from representatives who understand the complex demographic of the community. This was integrated with a range of techniques and approaches that may be of help with a condition characterised as a functional disorder and associated with fatigue, breathlessness, malaise and ‘brain fog’ and in many cases a reactive depression [1,4,5]. The design also sought to recognise and prioritise the needs and the potential benefits to the user group, reflecting those with low income, low employment status or retired, multi-cultural and household multi-generational or household isolated. Ethnic minority groups who are more likely to live in urban, more deprived communities and to work in lower paid jobs [29] have also been some of the worst affected by the pandemic [30].

The potential challenge of digital exclusion was addressed through an offer of support for digital devices and connectivity with personal support for any participants who required help. Participants were invited to complete the self-report on-line forms via a single link to their phone or computer and to attend three virtual clinic sessions using secured Zoom media. The Long COVID support sessions included a Sport and Exercise Medicine (SEM) doctor and either an occupational therapist, clinical psychologist or physiotherapist. The sessions were decided on availability of the research therapist/psychologist and the suggested need of the participant, based on the on line ‘modules’. The sessions explored the history of COVID-19 onset, diagnosis and experience of initial and then persisting symptoms. The SEM doctor screened for clinical signs or symptoms which indicated the need for NHS investigation and management. Conversations about pre-COVID physical activity levels and behaviours were facilitated and the impact of Long COVID on exercise tolerance and activity engagement was explored.

### 2.2. Implementation

The method of implementation of the virtual clinics is especially important where the target population are known to have lower engagement with health and social services. Previous negative experiences of healthcare services are known to inhibit future access to healthcare seeking behaviours [31]. In this case, the acceptability of the delivery of the pilot intervention was assessed through (a) the completion of the intervention by participants, (b) continuous reflection and adaptation by the multi-disciplinary team and (c) engagement of an expert stakeholder group involved in the co-production of the intervention.

The plan for recruitment included a specific identification process, followed by informed consent and recruitment, prior to the intervention. To identify participants, a local, community wellbeing group who partnered with the research team. The H&W worker used existing contacts in the local community and via a social prescribing network to identify those who had experienced COVID-19 infection and who continued to experience Long COVID type symptoms. The H&W worker was embedded in the community and so with the inclusion and exclusion criteria was able to contact and provide support to those who had so far not received any support for functional difficulties after more than eight weeks.

The inclusion criteria were adults, with self-reported COVID infection, with a minimum of six weeks recovery period. The area of recruitment was predicted to be culturally more diverse and potentially fewer people would have access to sufficient internet requirement. To address this potential barrier to recruitment the H&W worker was also able to accommodate some of these needs via language support and with use of a wi-fi enabled tablet device. Exclusion criteria were completely unable or unwilling to use a remote method to have contact with the research clinical team and those who ultimately on assessment were deemed to be at any risk of significant health or social problems that warranted further referral to GP and investigation.

The identification process involved the H&W worker approaching the participant (sometimes via referral from a third sector network) and offering an initial paper-based explanation of the purpose of the study and inviting participation. If the participant consented to further involvement their name, address and contact details (telephone and e-mail address) was passed to the project manager. The project manager opened and maintained a site file with personal information kept safely and separately from survey and outcomes of the intervention. The project manager began by distributing the participant information sheet and consent form that were embedded into the online survey tool. This tool enabled the content to be delivered via an e-mail link to a computer or mobile device that would be easy to text-read and required simple completion. The results are automatically uploaded to an anonymous portal that forms the basis of a minimum-data set related to each participant. Once consent was confirmed the project manager contacted the participant with a second weblink to the selected self-report questionnaires, when this was completed and returned a first contact date for a virtual meeting with the clinical team was arranged.

### 2.3. Data Analysis

The case report method [32] involves describing a group of patient participants who have a similar diagnosis and who negotiate a similar intervention over a certain period. It enables exploration of individual responses to an intervention to better understand who may benefit from it [32]. In this case, the common contextual factors and co-production of the intervention, resulted in several outcomes associated with (a) reporting clinical and functional problems from Long COVID and (b) the generation of theory to support the design of further studies. The needs of people who live in deprived communities are diverse and an individualised approach towards service development and implementation is pivotal [14]. There was no intention to make causal inference regarding the efficacy of the intervention but rather, and over a relatively short period with a small sample of participants, to generate an understanding of the opportunity to enhance access to care with a virtual involvement from the multi-disciplinary team. Furthermore, to learn from participants how their needs might be met with a brief intervention focussed on functional self-management.

Subsequent to all recorded meetings associated with each participant, the survey results, responses and reflections from the team were compiled into a series of ‘case notes’ to describe the intervention fully in each case and identify how the participant experienced the intervention and the degree to which they benefited. Using the Medical Research Council (MRC) framework [17] additional considerations included whether there had been any adverse effects known to have arisen from the intervention and whether the design of the intervention had, in a cost-effective way, increased access to care for a group of participants who had Long COVID but who had not access [8] care via the NHS. The ongoing potential of feasibility testing and implementing the study at scale was a key consideration with the potential of the method ultimately being adopted into practice, thus addressing the known problem of inequality of access for participants from more deprived groups.

In addition, and specifically because there is no known or accepted treatment pathway for Long COVID, the case studies were also analysed for common clinical phenomena and reported functional problems. This was done to understand the effect of the disorder in relation to physical and psychological wellbeing. Specifically, to describe general Long COVID characteristics related to person, place and time. The observational data collated during a case series is intended to describe the intervention, not to demonstrate efficacy [32]. The clinical materials reflect the focus of SEM that draws knowledge from physical activity as well as elite sport and treatment regimens that enable re-conditioning and also manage psychological effects of injury and infection.

Cross cutting inferences from the eight individual case studies were developed. By triangulating transcribed reports of the sessions, Qualtrics data on outcomes and case notes plus the care experience measure [26]. The research team completed a standardised proforma to combine the outcomes and experiences of the interventions including the participants own agency and motivations for small changes to their routine activities that would support a slow recuperation and restoration of health and wellbeing. This was also based on reflections and discussion at multidisciplinary team meetings to understand the Long COVID symptomology and condition including functional wellbeing and individualise the response in a short-term plan.

## 3. Results

### 3.1. Summary

Ten participants completed the pre-on-line self-report, and eight participants attended all three on-line interventions with the multi-disciplinary team and completed the post on-line self-report questionnaires. Two participants discontinued after session one and identified several critical issues including moving house (at short notice) and having other activity. Ethnicity was not directly sought but of the group four participants were white British with the others recognised as Asian and Black British. The virtual clinic meetings were delivered by arrangement in the participant’s home on Zoom, with two team members, a doctor and a therapist. An additional research team member managed the recording but did not engage in the session. The team aligned each session to the co-produced protocol devised to reflect clinical concerns and allow the participant to identify their main functional or symptomatic concerns. Acceptability of the intervention was based on the participant’s engagement with the service and the responses to the survey tools.

The first session involved finding out about experience of acute COVID-19 illness, social and medical history, and current problems. This was followed by an exploration of current physical activity levels and some initial advice that recognised the current level of physical and psychological tolerance and invited the participant to assess their activity and subsequent wellbeing. The second session involved the repeated sit to stand test for participants willing to engage in this. Verbal administration of the General Anxiety Disorder (GAD) and Patient Health Questionnaire (PHQ9) was completed as deemed appropriate by the clinical team with the option of continuing to record a wellbeing score each day. In some cases, a follow up email was used after each session, but this was not uniformly used with each participant. The final session took place within a negotiated time frame, sometimes with a delay of up to a month if the participant was able to prioritise their own recovery and activities within that time. The session included a reflection on their wellbeing and any progress or decline during the sessions and an invitation to complete the EuroQol and FSS assessment again with an addition Care Measure that acted as a participant reported experience measure (PREM). The follow-up arrangements were made typically including permission to contact the GP and finally personalised follow-up letter was sent from the research team with GBP 30 of vouchers in respect of their engagement. All sessions were recorded and stored securely within the online site file and in addition, all sessions were discussed in detail by the multi-disciplinary team during the recorded team meeting.

The research team also met twice with an expert steering group consisting of senior clinicians and people experience with Long COVID or fatigue symptoms. This knowledge exchange was a deliberate opportunity to continue PPI and work with colleagues who were delivering the NHS Long COVID clinics in the city. Although the project was not associated with the Primary Care organisation there were several references to the ways that participants had found it difficult to access their GP. It was notable that participants in the research-group had not presented via their GP to the Long COVID rehabilitation Hub and there was a belief that those from the more deprived areas were under-represented in the NHS clinic. The local Primary Care Network (PCN) was also represented in the steering group, recognising the potential need to refer participants to general practice for further investigation or onward referral Table 1.

Of these participants eight completed all the elements of the on-line questionnaires and the virtual clinic activity sessions. Details of self-reported pre and post intervention EQ-5D FSS scores are detailed in Table 2.

Inferential statistical analysis of self-reported data was not reflective of the aims of this project as the focus was on the acceptability of the co-produced intervention and experiences reported. Descriptive interpretation of the self-reported EQ-5D and FSS scores indicates that most participants experienced some reported improvement in health-related quality of life and fatigue symptoms, notably in mobility and ability to sustain usual activities. In some cases (P5,P6), the scores indicated a slight increase in symptoms, the case descriptors below further explore these trends.

The scores recorded for the CARE Measure indicated that the participants had felt listened to and supported by the clinical team, the responses are detailed in Table 3.

### 3.2. Case Descriptors

A brief case description of each active participant is provided below with a more extended version provided in Supplementary Materials. The information includes demographic detail that suggests participant’s household status and range of problems associated with Long COVID. The narratives are a snapshot of the participants who completed the intervention and completed the outcome and experience data.

Participant One was a 41-year-old South Asian British man experienced diffuse pain symptoms, fatigue and headaches. *‘It’s like I’ve still got COVID inside me, it’s like it’s not gone.’* He had high levels of pain, anxiety and fatigue which had reduced his working day as a taxi-driver to only 1–2 h meaning that he was no longer the breadwinner for the family. His agreed plan included short walks and careful monitoring of post exercise malaise. During sessions he travelled to his country of origin but continued to progress and added small weights to his walking pattern, being unable to return to the UK due to quarantine restrictions. His outcome scores demonstrated improved mobility but continuing anxiety. He said *‘Everything is still different, because of COVID, work and family life is different. Until everything returns to normal I cannot know what can and cannot be done.’* His experience of the intervention was very positive and his self-report module scores reflected perceived improvement in pain and fatigue symptoms, although he reported feeling unable to plan further actions.

Participant Two was 38-year-old East European British woman who lived with her husband and three school age children and was in part time employment. She reported Long COVID symptoms including headaches, palpitations, bruising and fatigue that dramatically reduced her activity levels; even a walk to the nearby school left her feeling very tired and breathless. She described a constant level of stress ‘*Three kids, work, home schooling and trying to work from home…..it’s really difficult to juggle everything.’* The intervention introduced a short walk and adoption of pacing principles to manage bouts of activity and rest. In the sessions she was able to share her difficulties associated with self-confidence. Outcomes included reduction in pain and marked improvements in self-reported motivation and energy for family, work and social life *‘Sometimes at my desk I would think ok, I need to move, and I’d set the timer and do the sit to stands….it felt good.’* Her experience of the intervention was universally excellent.

Participant three was a 75-year-old retired white British man who lived alone after a recent divorce prior to lockdown which he had found lonely and difficult. He had several co-morbidities including psoriasis, arthritis, tinnitus and depression. His self-reported quality of life demonstrated an ability to cope but this was impacted by fatigue symptoms affecting motivation and sleep disruption; ‘*I nod off and then it’s teatime and then I can’t sleep at night.’* The intervention included a ten-minute health walk with support for breathlessness, joint pain and fatigue. He bought a pedometer and used a spreadsheet to monitor progress, adding a British Heart Foundation exercise video to his daily regime. During the sessions he shared more personal information about restlessness, anxiety, and guilt about his marital breakdown. During the final session he stated that he felt ready to return to a group health walk; ‘*I can’t wait to get started, when they open things up again I want to get going.’* His outcome scores demonstrated re-engagement with usual activities and his response to the intervention was excellent.

Participant Five was a 75-year-old white British woman who was a retired factory worker. She lived alone with nearby family and had pre-existing COPD. She reported difficulties with self-care and anxiety and high fatigue. She was affected by the death of her husband from a brain tumour four years ago and as well as losing her spouse she had also lost her social group, reduced still further due to a friend’s death from COVID. She described ‘*I don’t see nobody, I’d just like to get on the bus and go into town and see someone.’* Lockdown had an increasingly sedentary effect on her lifestyle that appeared to exacerbate her already existing symptoms including a cough. Her report following the intervention demonstrated that her awareness of her limitations had increased and was worse for pain and mobility. Whilst she reported an excellent experience of the intervention, the team were conscious of her need to further engage with a social group such as offered by the local Wellbeing group.

Participant six was a 45-year-old white British woman who lived with two disabled sons and who also supported her elderly mother. Her symptoms included fatigue that severely affected her routine and exacerbated asthma. She also described having flashbacks to the acute phase of the viral illness likened to post traumatic stress disorder (PTSD). She described pain and anxiety which had caused her to limit all activity to a two-hour ‘window’ each day and also articulated frustration with living in a polluted environment. She was very aware of disability and racial rights and conscious of significant prejudice when using public transport with her sons. With very limited support she had withdrawn socially and resigned to having very limited physical and emotional energy, referring to Long COVID support as ‘*poking in the dark, not knowing what will help people’.* Given a clear reluctance to engage in physical ‘tests’ that she regarded as demeaning; *‘I’m not a performing monkey.’* She did identify a need for more social contact, accessing green spaces and maintaining standards with home and family life. The focus of the intervention was to legitimise rest and relaxation, pacing and sometimes taking the easier option, for example, catching an Uber instead of the bus. Her feedback was that this was a process of *‘Learning about yourself.’* with limited improvement scores in self-care and anxiety and feedback suggesting a greater emphasis on taking control and planning action.

Participant seven was a 53-year-old Muslim woman who had moved to the UK from the Middle East approximately 15 years earlier. Now divorced, she lived with her four teenage children and was an active member of the community and enrolled in an MSc in computing. To ‘*get back to normal’* following COVID-19 infection she had fallen off her bike and fractured her humerus, she also reported struggled with fatigue, poor concentration and had a sensation of heavy weight on her chest and night-time breathlessness. With pre-existing diabetes and osteopenia, she reported high ‘anxiety’ and severe limitations to family activities *‘We’d always go on outings, to the seaside, the park. Get them out in the fresh air.’* Since COVID she becomes tired after walking 20 min with recent weight gain and occasionally overeating and craving carbohydrates. She also described musculoskeletal pain from joint and tendon niggles. During session two she shared more about her past which included an abusive relationship with her husband and loss of her father in 2020. Her plan included daily short regular walks with pacing and rest planning and referral to the GP for support with menopausal symptoms. Her response to the interventions was summarised by this comment *‘for the first time I felt like I mattered, to have two professional women listening to me, understanding me,’* and her post intervention scores demonstrated an improvement in all areas.

Participant nine was a 61-year-old white British woman who lived alone and experienced worsened breathlessness and fatigue with an early diagnosis of COPD, a history of heavy alcohol use and chronic low back pain. She was recently divorced from an abusive relationship and bereaved following the death of her mother, to whom she was very close. She reported pain and fatigue and she shared that her confidence had been eroded in the relationship with her ex-husband and feeling very worried about crowded spaces or travelling to see sons who did not live nearby. She engaged with anxiety management advice and was encouraged to manage breathlessness and during the period took a short holiday to meet her family. Clearly motivated by these achievements she said, ‘*I’m seeing things differently, talking to you two has helped me, soothed things a bit, calmed the waters.’* She reported improvements in anxiety and usual activities and slightly improved fatigue and an excellent experience of participating.

Participant ten was a 38-year-old Black British woman who lived with her two school age children and was employed as a nurse in the NHS. Whilst she had tried to return to work, breathlessness and fatigue meant that she could not sustain her previous commitment to work as she had to prioritise with her childcare commitments. Being active and using exercise to manage weight, she had felt too fatigued to take her children out and was tearful when describing the constant imperatives of her life: *‘always having to get on, I can’t really rest.’* Her plan included increased physical wellbeing and weight loss to lower her blood pressure and prevent onset of diabetes and she engaged with a stair walking programme combined with relaxation techniques. She also started to walk part of the way to school and had taken the children to the park. She explained that she rarely opens up to people and felt a sense of relief for having talked about her mood and challenges she faced. She commented *‘I’ll remember you guys, thank you, thank you, for all you’ve done.’* Her fatigue reduced and her engagement experience was excellent.

### 3.3. Cross Cutting Inferences

#### 3.3.1. Temporal Changes and Self-Reflection

All participants reflected on changes to their lifestyle attributed to COVID. Although most participants experienced hardship and challenges prior to the onset of COVID, they recalled active routines with a sense of fulfilment and purpose associated with their self-identity. Knowledge of health behaviours was evident and a number of participants had previously committed to positive changes including smoking cessation, alcohol and weight management. The sentiment of ‘never being an athlete but I tried to be active’ was shared by several participants when recalling their pre-COVID lifestyle. All participants described a more sedentary way of life following COVID infection, primarily attributed to their physical symptoms and compounded by the lockdowns. Breathlessness, fatigue and pain were commonly reported symptoms which deterred engagement with physical activity. During the clinic sessions, participants shared insights into their psychological status with loss of confidence and poor self-efficacy emerging as recurrent sentiments. Some participants shared that they had experienced long term challenges associated with their self-esteem and mood. The experience of living with Long COVID had heightened these pre-existing challenges to a point where they presented barriers to participation in social interaction and a functional standard of living. Engagement with the SEM virtual clinic facilitated reflection on self-identity and in most cases empowered participants to recognise that they could access support to rebuild their social network and routines. It was identified that on-going support would be beneficial for participants to continue with their recovery.

#### 3.3.2. Under Reporting of Differences between Men and Women

Eight participants who consented to the project were women. Analysis of the case notes and recorded sessions detected some differences associated with participant gender. The two male participants adopted a quantified approach towards their resumption of physical activity, for example, they reported exact step count achieved on walks and aimed to progress their performance through increased duration or intensity of activity. The female participants focussed more on how they felt during activity and the integration of exercise with daily routines and family commitments. Several female participants reported recent changes to their menstrual cycle which may have been indicative of peri-menopause or menopause. This compounded the symptoms experienced, for example, brain fog, sleep disruption and fatigue, which can be associated with menopause or Long COVID. In some cases, the exploration of these symptoms during the virtual clinics triggered access to GP services for assessment of hormonal status.

#### 3.3.3. Personalised and Acceptable Clinical Intervention 

The total duration of the three sessions ranged between 2–3 h for each participant and yet the exercise prescription comprised short walks and the repeated sit to stand exercise. The complexity of the conversations developed as participants gained trust in the clinical team and shared personal and sensitive information relevant to their physical and psychological health. The prior lack of engagement with health services for COVID or long COVID was notable in the group of participants and one of the most reported benefits was simply talking to a trusted professional. Reflection upon their experience of COVID, pre-COVID lifestyle and current circumstances opened up recent and distant memories. Barriers to engagement in physical activity and social participation were explored and the clinical team were required to draw upon their expertise to frame the intervention in terms which were acceptable for everyone. Although the team shared a common protocol to guide each session, the execution of each contact was unique and guided by the participant. The clinical team applied their clinical judgement to determine which tests and activities were indicated for each participant to ensure a tailored and individualised programme of care.

#### 3.3.4. Scoring and Changes in Health Status

Among the eight participants there was a tangible level of improvement that was reflected in the pre and post scores. The bespoke intervention was regarded as an opportunity to discuss significant difficulties encountered during and since the COVID infection and most notably fatigue, pain and anxiety scores improved. The careful attention received by participants was reflected in several comments suggesting that people felt their needs were ‘worthwhile’ and that they had benefited from engagement with a ‘trusted professional’. Whilst the virtual nature of the encounter may have been a barrier to some, the feedback from participants suggested that the attention to their needs and the intensive input was strongly validating of them and their current situation and this is evident in the early data on effectiveness.

## 4. Discussion

The aim of this project was to co-produce and implement an intervention for people with Long COVID, living in communities recognised as more deprived and evaluate its acceptability. The recognition of being more deprived and ethnically diverse was based on the IMD [24] that allocates households to Lower Layer Support Output Area (LSOA) around 1500 households in each. The research sought to investigate the experience of Long COVID and to work closely with participants to produce an acceptable virtual intervention. The intervention was delivered during a lockdown period and through a digitally enabled intervention which utilised on-line data collection and reporting, in combination with virtual meetings using Zoom (Zoom.us) The partnership with the pre-existing and locally trusted community organisation enabled those with sufficient English language and with sufficient on-line ability to take up the offer. By managing digital exclusion, the on-line requirements became an opportunity for the very fatigued to take part. The recruitment strategy was an important element of the learning; assertively facilitating participation via a H&W link worker who could provide hardware or personal wi-fi access as well as encouragement and personal support.

The co-production process for an acceptable intervention for Long COVID symptoms was significant because it has been recognised that people from diverse ethnicity heritage experience barriers to the take up of services for long term conditions [33]. The research was associated with the development of ‘public health assets’ that are more likely to address health inequalities [34] and to innovate in implementing community practices. The virtual clinic modelled a bespoke intervention for Long COVID, using a multidisciplinary team to enable an individual to plan and consider their personal assets and capabilities [35]. The team intervention recognised the multiple and complex influences on an individual (neuromuscular, biomechanical, sensorimotor and neurocognitive) that factor in the process of optimising their recovery. This rehabilitation approach was novel insofar as sport and exercise medicine had not been included in this way and the focus on performance enhancement was valued by participants. However, it is important to recognise that ‘health assets’ such as virtual clinics, particularly where participants mainstream clinical services cannot be uncoupled from the wider community sustainability agenda [36].

The intervention was characterised by compassionate listening and attending to the needs of an individual and a careful attention to the interests of that person in their own context. This approach is reminiscent of health coaching in which the clinical teams partner with patients to enhance self-management strategies for the purpose of preventing exacerbations of chronic illness and supporting lifestyle change [37]. Whilst the health coaching modality is under researched in terms of effectiveness, there is some evidence that personalised goal setting, supportive communication associated with motivation, and collaboration with other health care providers and assets (i.e., exercise venues and trainers) benefits some people [38]. The most recent literature associated with health coaching focuses on psychological, physiological and behavioural combined outcomes [39] although also challenges the idea that it is effective in improving quality of life or improving mood [40]. Telehealth methods and apps are also deployed recently although the comparison between benefits of person-to-person peer coaching verses remote or app-based support, has not been substantiated [41].

In this study, the team, made up of SEM doctors, physiotherapist, occupational therapist and psychologist provided a high level of input over three sessions. Findings, based on a small sample and specifically targeting support to an under-served community suggested that careful consideration needs to be given to individual and personal factors associated with gender and household responsibility and that the team focus needs to be on current health status. In this case, the inclusion of medically trained professionals provided some reassurance to the team. Individual participants directed the intervention in relation to simple and focused activities that were possible to arrange and incorporate in their personal routine. The focus on routine and attention to what could be achieved was important as was the need to apply simple techniques to the manner in which their routine was managed (i.e., pacing methods, step counting, sit to stand set monitoring). Exercise in this case was not an outcome but rather a means of achieving a more considered approach to wider wellbeing.

In elite sports medicine, the ambition is often to enhance athletic outcomes and performance but SEM can work in a general population setting as well as in an elite/athletic settling when based in a clinical team and where there is a shared focus on person centred care. Health coaching being a shift in focus within traditional NHS services and becoming more obviously needed with people experiencing post COVID symptoms. The agency of the participant is the key factor in participation and engagement, the end users being the decision-maker in the process of rehabilitation [42]. The participation in clinical academic teams is showcasing the importance of integrative knowledge and the need to apply techniques to the context of the individuals experience, working with a realist understanding the context–mechanism–outcome relationship, by considering their interactions [43]. This co-produced intervention included multiple elements and providers which were tailored to the target population. This represents a challenge in terms of replication of the delivery model across different patient populations and medical infrastructures although comparable community partnerships models to deliver complex health interventions have similarly met the needs and preferences of service users [16]. Ultimately, the wider adoption of asset-based collaborative community services is dependent on local policymakers [37].

In this study, the identification and recruitment of participants who may be ‘harder to reach’ was a deliberate strategy, linked to the primary aim which was to co-produce an acceptable intervention for people with Long COVID, living in communities recognised as more deprived. Intrinsic and extrinsic barriers to engagement in positive health behaviours amongst deprived populations have been previously reported and this project elucidated comparable findings [44]. The participants were aware of the inter-relationships which existed between their wellbeing and modifiable behaviours including diet, physical activity and sleep patterns. The co-produced intervention facilitated identification of small changes which could be realistically introduced into daily routines to enable participants to gradually increase their activity levels within the limits of their Long COVID symptoms. The importance of monitoring symptoms was emphasised and participants were advised to contact their clinical team if an exacerbation occurred between sessions.

Acceptability of interventions is a Medical Research Council recommendation for complex interventions in which context is all important. It includes the wider socioeconomic background (including underlying cultural assumptions), often reflecting the fidelity of the delivery and the characteristics of the participating population. In this case, the prevalence or severity of the condition studied was somewhat unknown and it was uncertain whether and how the intervention would be received given the complex social and historical events compounded by lockdown. Key domains of importance as a consideration of the acceptability in this case appear to be associated with (a) the identification of simple solutions and ‘small’ support that enabled local activity and (b) highlights the importance of being heard by a ‘trusted professional’ in the context of a clinical intervention [45]. The holistic approach, which extended beyond the immediate symptoms of Long COVID and engaged with historical bereavements, traumatic experience and existing pressures and imperatives associated with family, demonstrate that health coaching extends into advocacy and may increase participants’ perceived value of the service. Eight out of ten recruited participants completed all three sessions; the two participants who discontinued reported less severe symptoms at the outset of the project which may indicate the perceived value of or need for the service was lower for those individuals. This work concurs with similar international literature that suggests that the intervention included other aspects of benefit to the participant, such as advocacy and supporting increased participant control of health all within a culturally appropriate coaching model [46].

### Strengths and Limitations

In this project the participants were not engaged in planning the project and this was a limitation. However, the research included collaboration with the trusted 3rd sector partners and a preliminary public engagement group, with experts identified for their ability to advise on needs expressed by diverse ethnic groups. They included those with health problems associated with long COVID, i.e., those with chronic fatigue and pain and those from the specific targeted area of deprivation. Recruitment for phase two of the project could have been more precisely quantified through an audit trail of the number of people approached compared with uptake and consent. The study was consultative and reports findings but could be extended to include further and ongoing participation in a continuing case series.

A group of social care practitioners were also included for their experience as social prescribers in more deprived communities. This preliminary expert co-production process enabled the conceptual framing of the intervention, ensuring that known barriers to engagement of underserved communities were managed, for example, identification and recruitment by a trusted local link worker and facilitated IT were critical.

The outcomes of the intervention, whilst demonstrating an early and positive trend associated with the participants self-rating of fatigue and quality of life, this is not a reliable indication of efficacy.

Self-management of Long COVID via person-centred care and health coaching requires further substantiating in more deprived communities to attend to the cultural and contextual factors associated with barrier to engaging in mainstream health and care.

## 5. Conclusions

This study developed an intervention that aimed to reduce the effects and support self-management of Long COVID symptoms in a deprived area of Sheffield UK. The clinical academic team combined professional disciplines and shared an approach that enabled enhanced physical performance. The planning of the intervention incorporated a number of benefits associated with health coaching including advocacy, additional social and psychological support and enabling self-management. Significantly, the use of PROM and PREM data enabled the value of the intervention to be assessed alongside the other factors associated with recruitment and retention to the research. Participants valued the tailored and individualised clinical delivery model and engaged with the intervention. The individualised approach empowered participants to adopt small but effective health behaviour changes which were associated with improvements in reported wellbeing. Further study is needed to identify the cost and viability of virtual clinical interventions for long COVID in underserved communities.

## Figures and Tables

**Table 1:** Participants’ demographics and functional status.

Participants	P1	P2	P3	P4	P5	P6	P7	P8	P9	P10
Age	41	38	75	47	75	45	53	48	61	38
Sex	M	F	M	F	F	F	F	F	F	F
IMD ****** Decile	2	2	2	1	1	2	1	1	1	1
Employment status	FTSE *	PTE **	Retired	UE ***	Retired	Unpaid Carer	UE ***	UE ***	Not stated	PTE **
Household status	MG ****	Spouse plus DPS	Lives Alone	Spouse	Lives Alone	DPS *****	DPS *****	Spouse	Lives Alone	DPS *****
Post COVID Functional Status Scale	2	1	2	1	2	3	3	3	3	2
Project Completion	Yes	Yes	Yes	No	Yes	Yes	Yes	Yes	No	Yes

FTSE * (full time self-employed), PTE ** (part time employed), UE *** (unemployed), MG **** (Multi-generational), DPS ***** (dependents), IMD ****** (Indices of Multiple Deprivation)

**Table 2:** Pre and post intervention EQ-5D and FSS responses.

Participants	P1	P2	P3	P4	P5	P6	P7	P8	P9	P10
Pre-intervention EQ-5D Mobility	2	1	3	1	2	3	3	3	3	2
Pre-intervention EQ-5D Self care	1	1	2	1	1	3	2	1	3	1
Pre-intervention EQ-5D Usual Activities	3	2	3	1	2	NC *	4	2	4	2
Pre-intervention EQ-5D Pain	3	3	3	2	2	4	3	2	4	2
Pre-intervention EQ-5D Anxiety	3	1	3	3	1	5	5	2	4	2
Pre-intervention EQ-5D Total	**12**	**8**	**14**	**8**	**8**	**15**	**17**	**9**	**18**	**9**
Pre-intervention Fatigue Severity Score	**51**	**35**	**52**	**32**	**46**	**63**	**58**	**52**	**62**	**33**
Post-intervention EQ-5D Mobility	1	1	3		3	3	2		3	1
Post-intervention EQ-5D Self care	1	1	2		2	4	3		3	1
Post-intervention EQ-5D Usual Activities	2	2	2		2	4	3		3	1
Post-intervention EQ-5D Pain	2	2	3		3	4	2		4	2
Post-intervention EQ-5D Anxiety	3	1	3		2	4	3		3	2
Post-intervention EQ-5D Total	**9**	**7**	**13**		**11**	**17**	**11**		**16**	**7**
Post-intervention Fatigue Severity Score	**32**	**28**	**37**		**55**	**61**	**54**		**59**	**22**

NC * (Not completed), bold font indicates total scores for each scale. The Euro-Quality of Life-5D—EQ-5D.

**Table 3:** CARE Measure responses.

Participants	P1	P2	P3	P5	P6	P7	P8	P10
Making you feel at ease	5	5	5	5	4	5	5	5
Letting you tell your ‘’story’’	5	5	5	4	5	5	5	5
Really listening	5	5	NC *	5	5	5	5	2
Being interested in you as a whole person	5	5	5	5	5	5	5	5
Fully understanding your concerns	5	5	5	5	4	5	5	5
Showing care and compassion	**5**	**5**	**5**	**5**	**5**	**5**	**5**	**5**
Being positive	**5**	**5**	**5**	**5**	**4**	**5**	**5**	**5**
Explaining things clearly	5	5	5	5	4	5	5	NC *
Helping you take control	5	5	5	5	3	5	5	5
Making a plan of action with you	4	5	5	5	3	5	5	5
Total	**49**	**50**	**45**	**49**	**42**	**50**	**50**	**42**

NC * (Not completed). Bold font indicates total scores for each scale.

## Data Availability

Not applicable.

## Supplementary Materials

The following are available online at https://www.mdpi.com/article/10.3390/ijerph182413191/s1, Extended Case Description.
