# Supplementary material for: Assessing the Acceptability of a Co-Produced Long COVID Intervention in an Underserved Community in the UK
Source: Int J Environ Res Public Health. 2021 Dec 14;18(24):13191. doi: 10.3390/ijerph182413191 (PMC8701839; doi:10.3390/ijerph182413191)
Supplement: Supplementary file 1 [file ijerph-18-13191-s001.zip › ijerph-1421333-supplementary.pdf]

Supplementary materials – Extended Case Description
Participant One

Participant 1 was a 41 year old male who reported that he thought that he caught Covid-19 through his work as a taxi driver in March 2020. His main symptoms were a dry cough, chest tightness, expectoration and breathlessness which persisted for 8 weeks. He was not tested for Covid-19 and managed his symptoms at home. The symptoms never fully resolved and evolved into unexplained diffuse pain symptoms, fatigue and headaches. He underwent NHS investigations including scans and blood tests which detected low levels of vitamin D but no pathology. When asked about his perception of the cause of his symptoms he stated *'It's like I've still got Covid inside me, it's like it's not gone, all these symptoms.'* At the start of the project his self reported EQ-5D5- score was 12 associated with high levels of pain and anxiety. His FSS was 51 and he reported that due to fatigue he had reduced his work commitment as a taxi driver from 8 hour shifts to a maximum of 1-2 hours per day. He had also stepped away from family and household duties and he felt that his identity as the family breadwinner had altered.

Prior to Covid-19 he had enjoyed going for walks when the weather was good; he could easily manage 10 000 steps and usually felt refreshed afterwards. During the first session he reported that he now experienced difficulty walking 5000 steps and would need to rest for several hours afterwards. During session one he was advised to aim to walk shorter distances on most days, 2000-3000 steps and to monitor his fatigue through daily wellness scoring (DWS). He engaged well with the DWS which indicated a fluctuating but overall improving pattern of symptoms. During sessions two and three he was based in Pakistan where he was accompanied by his wife who was accessing health care there. Repeated sit to stand was introduced during session two and he verbally reported that he had engaged with this. During session three we suggested that he could progress this exercise by wearing a rucksack with 2KG. During session three, P3 was asked about his plans for returning to work and family life commitments. He responded *'everything is still different, because of Covid, work and family life is different. Until everything returns to normal I cannot know what can and cannot be done.'*

On completion of the project his EQ-5D5-5L score was reduced from 12 to 9 with improvements in self-reported mobility, usual activities and pain. His anxiety score was unchanged at 3/5. His FSS had reduced to 32 with the most marked self-reported improvements in the association between fatigue symptoms and his ability to engage in exercise and family or social activities. His PREM evaluation scored fives on all components except 'plan of action' which was scored as 4/5.

Participant Two

P2 was a 38 year old female who lived with her husband and three school age children. She reported a two day illness in March 2020 during which she felt very tired and unable to get out of bed. She was not tested for COVID-19 as it was prior to availability of mass testing. However, she experienced new and sustained symptoms following the viral infection similar to those now associated with Long COVID including headaches, palpitations, bruising and fatigue. She underwent some investigations via her GP which included ECG and blood tests.

She also accessed a chest X-ray privately. Prior to Covid she was conscious of the importance of exercise and aimed to walk 10 000 steps per day, but fatigue symptoms reduced her current activity levels and she reported that even the short walk to school left her feeling very tired. She described becoming very breathless on any activity which involved lifting and carrying, for example gardening. She described a constant level of stress and was also medicated for hypertension. When asked what makes her life stressful she responded '*three kids, work, home schooling and trying to work from home…..it's really difficult to juggle everything.'*
Her total EQ-5D-5L score at the start of the project was 8, with pain being the highest scoring domain. The FSS at the start of the project was 35.

The intervention introduced to P2 comprised walking a short distance every day, repeated sit to stand and adoption of pacing principles to manage bouts of activity and rest. Her sit to stand score increased from 14 reps to 20 reps in 30 seconds over a two-week period. During sessions two and three she increasingly opened about confidence issues which stemmed from the relationship with her mother. She was quite tearful during these conversations and counselling to explore and access support for this was recommended. During session two we also went through the GAD7 and PHQ9 questions. Her scores were PHQ9=10 and GAD7=9. During session three she talked about the repeated sit to stand, she commented *'Sometimes at my desk I would think ok, I need to move, and I'd set the timer and do the sit to stands….it felt good.'* On completion of the three sessions her EQ-5D-5L score was 7, with pain score decreased from 3 to 2. The FSS decreased to 28 with marked improvements in self-reported motivation and energy for family/work/social life. She scored 5/5 on all domains of the PREM.

Participant three

P3 was a 75 year old retired male who lived alone. He was recently divorced and has two grown up children. He has sustained an amicable relationship with his ex-wife and found living alone during the lockdowns lonely and difficult. He reported a viral illness in March 2020 with symptoms of COVID-19 including loss of taste/smell, breathing problems and ached all over. He stayed in bed for the first few days and then gradually improved. He was not admitted to hospital or tested. Since the infection he has felt more breathless and fatigued. He had several co-morbidities including psoriasis, arthritis, tinnitus and depression. His methotrexate prescription for psoriasis was stopped at the start of the pandemic but he was prescribed a statin for high cholesterol and sertraline for depression which also helps with the tinnitus. At the start of the project his EQ-5D-5L was 14 with 3's scored across all domains apart from self-care which was 2/5. The FSS at the start of the project was 52 with very high scores for the impact of fatigue symptoms on motivation and exercise. He described sleep disruption associated with fatigue; '*I nod off and then it's teatime and then I can't sleep at night.'*

He recalled being an active member of Darnall Wellbeing and used to enjoy their weekly health walk, he felt that he would struggle to manage this now due to breathlessness, joint pain and fatigue. During session one we introduced the goal of a 10 minute walk each day. P3 engaged with this and bought a pedometer to record his daily distance and step count which he shared during session two on a spreadsheet he had created. During sessions two

and three he sustained engagement and progressed to add repeated sit to stand plus and BHF exercise video to his daily regime. He experienced a couple of minor setbacks including shoulder pain associated with the second vaccine and increased fatigue when his methotrexate prescription was re-started. With guidance he adjusted his exercise programme according to his overall wellness on a given day. During session two we talked through PHQ/GAD and he shared that he tends to be restless and anxious and feels guilty about his marital breakdown. However, he wanted to carry on and build a good life ahead for himself. He indicated that he would like to meet someone and had thought the virtual sessions may have been a group forum. During the final session he stated that he felt ready to return to the Darnall Wellbeing health walk, stating; '*I can't wait to get started, when they open things up again I want to get going.'* His repeated sit to stand score increased from 14 to 21. On completion of the three sessions his EQ-5D-5L score was 13, the improvement being in self-reported usual activities. The FSS decreased to 37 with improvements in all domains. He scored 5/5 on all domains of the PREM apart from 'listening' which was blank.

Participant four (5)

Participant four was a 75 year old female who was retired factory worker who lived alone. She is an ex-smoker and lives with a diagnosis of COPD. She reported that her COVID-19 symptoms started on 24/12/20 and on 03/01/21 she received a positive test result. She felt extremely unwell during the acute phase of her illness and had a severe cough. She thought she might die but did not contact her GP and remained at home throughout the illness. She felt that she had never fully recovered to her pre-COVID level of health with continued productive cough, fatigue and breathlessness. At the start of the project her EQ-5D-5L score was 8 with scores of one for self-care and anxiety. Her FSS score was 46, however, two items were missing and she had indicated 5-7 on all domains indicating high self-reported fatigue.

During the sessions she shared a narrative of loss starting four years earlier with the death of her husband from a brain tumour. They had socialised together and her social circle has changed since the bereavement. She lost a friend to COVID-19 in January 2021 and experienced an unpleasant fall out with another close friend shortly before COVID lockdown. She used to go to town centre every day, but since COVID this has not been possible. She does have children, grandchildren and a great grandchild nearby. She has a smart phone but does not use the internet and prefers to speak on the phone to maintain contact.

She described feeling lonely and lazy. During the first session she stated '*I don't see nobody, I'd just like to get on the bus and go into town and see someone.'* Since COVID she has been doing less housework and walking less. The productive cough was identified as her most problematic symptom and a key reason for her increasingly sedentary lifestyle. She has several regular prescriptions for inhalers and blood pressure medication but limited connection with her GP services as she felt like she no longer knew the GP's. The interventions recommended by the project team included regular walking, sit to stand, chest X-ray via GP, resume visits to town when restrictions ease and contact DWB for longer term support. During the project she reported that she was taking longer walks and had

booked a winter holiday. She engaged in the sit to stand exercise during sessions two and three.

At the end of the project her EQ-5D-5L score had increased to 11, with worsened self-reported mobility and pain. The team felt that the conversations focussed on her health may have increased her awareness of her limitations which may have contributed to the change in her self reported QOL. Her FSS scores were very similar to baseline, exact comparison is not valid due to missing data components. The team felt that she had benefitted from being able to talk at her own pace and level of detail during the clinic sessions. She scored 5 across all domains of the PREM with the exception of 'story' which was 4/5.

Participant five

Participant five was a 45-year-old female who lived with two 20-year-old sons who were dependent on her due to severe autism and also supported her elderly mother who lived nearby. She reported contracting COVID-19 in March 2020 and described a severe acute illness during which she spent several days on the floor with loss of smell and raised temperature. She was not admitted to hospital and developed long term symptoms including fatigue and exacerbated asthma. She described having flashbacks to the acute phase of the viral illness likened to PTSD. The fatigue symptoms were severe and profoundly affecting her routine; she identified a two-hour window each day during which she could engage in cognitive or physical activity. At the start of the project her EQ-5D-5L score was 15 (not factoring for a missing dimension for 'self care) with scores of four and five for pain and anxiety respectively. Her FSS score was 63, scoring 7's for all dimensions.

She described feeling that she was a mentally strong person but the accumulative effect of caring for her sons and mother, living in a polluted environment, perceived lack of support and Long COVID symptoms caused her to become frequently tearful. She has been prescribed fluoxetine and referred to STH Long Covid Hub but described this as '*poking in the dark, not knowing what will help people.'* Prior to COVID-19 infection she was an active member of the community and held several non-executive roles in the local schools and NHS Trust. She was employed at CAB and a senior advisor for benefit appeals. She was very aware of disability and racial rights and conscious of significant prejudice when using public transport with her sons. She has withdrawn socially and resigned to having very limited physical and emotional energy.

She was willing to engage with the project and wanted to support it as a participant. However, her view of supportive interventions was cautious and stated that she '*didn't want to waste her time with Billy Graham type video chat.'* She declined doing the sit to stand test, stating that she was not a *'performing monkey.'* The clinical team focussed on building rapport, sharing concerns and enabling a sense of control by asking "what will help you?" Although initially unsure of how to respond to this question, she did identify solutions which she felt would enhance her wellbeing which encompassed making social contact, accessing green spaces and maintaining standards with home and family life.

The sessions appeared to facilitate reflection on her Long COVID and she identified it as being a process of *'learning about yourself.'* The team helped her to legitimise rest, pacing and sometimes taking the easier option, for example, catching an Uber instead of the bus. At the time of the project, she was very isolated, but during the final session she expressed that she would like to engage in peer support activities and follow up through DWB. At the end of the project her EQ-5D-5L score was 17, however, on this occasion she had completed all sections and reported improvement in self-care and anxiety. Her FSS score had reduced slightly from 63 to 61 with improved reported ability to exercise. Her PREM scores spanned between 3-5 with fives for story, listening, whole person and compassion; threes for taking control and plan of action.

Participant six

P6 was a 53-year-old Muslim woman who had moved to the UK from Syria approximately 15 years earlier. She was divorced and lived with her four teenage children. She was an active member of the community and enrolled on an MSc in computing. She reported that she had tested positive for COVID-19 in July 2020 and symptoms at the time included a sore throat, headache and loss of taste and smell. She was not admitted to hospital and approximately one month later had attempted to '*get back to normal*' by going for a short bike ride to meet some friends for a picnic. She had fallen off her bike and fractured her humerus. She has since struggled with fatigue, poor concentration and had a sensation of heavy weight on her chest. She experiences breathlessness which is worse at night. P7 had a few co-morbid health conditions including diabetes and osteopenia. At the start of the project her EQ-5D-DL score was 17, with highest scores on the 'anxiety' and 'usual activity' domains. Her FSS scores were also high with a total of 58.

Prior to COVID she described an active lifestyle. She felt that being active with her children was important and stated *'We'd always go on outings, to the seaside, the park. Get them out in the fresh air.'* She went for regular walks and to the swimming pool once a week with her daughter to do aqua aerobics and then a lane swim; *'I was never an athlete but knew I had to move and exercise.'* Since COVID she becomes tired after walking 20 minutes and felt that she had to do everything slowly; *'my kids call me tortoise, even my driving is like a snail.'* She reported recent weight gain and a difficult relationship with food, occasionally overeating and craving carbohydrates. She also had musculoskeletal pain from joint and tendon niggles. During session two she shared more about her past which included an abusive relationship with her husband and loss of her father in 2020. Her PHQ score was 20 and she contacted her GP for support for her mood.

P7 engaged in open conversation during the sessions and her intention to become more active was clear. The team advised about short regular walks with pacing and rest and she engaged with this. Some of her symptoms including sleep disruption, weight gain and poor concentration may be associated with Long COVID and menopause. She reported her periods had stopped one year earlier and on advice from the team contacted her GP regarding hormone assessment and HRT. She missed one session due to over-sleeping and was upset about this, writing in an email afterwards; *'for the first time I felt like I mattered, to have two professional women listening to me, understanding me.'* At the end of the project her EQ-5D-DL scores was 11, with improvements reported in all domains. Her FSS

score was also decreased to 54 with exercise the most marked area of improvement. She scored 5 ‘excellent’ on all domains of the PREM.

Participant seven

Participant seven was a 61-year-old Caucasian woman who lived alone and was tested COVID-19 positive on 6th January 2021. She was very unwell for four weeks with cough, fever, aches and fatigue. She was bedbound but did not seek NHS treatment and has continued to experience worsened breathlessness and fatigue. She now needs her daughter to help with bathing and can only walk her small dog short distances. She was a light smoker with an early diagnosis of COPD. She also had a history of heavy alcohol use and chronic low back pain. She was recently divorced from an abusive relationship and bereaved following the death of her mother, to whom she was very close, two years earlier. At the start of the project her EQ-5D-5L score was 18, with pain, anxiety and usual activity domains all self-reported at 4/5. Her FSS score was 62.

During the sessions her breathlessness at rest was evident and during completion of the GAD-7 (16/21) and PHQ-9 (15/27), she shared that she had lost her confidence. She recalled being an outgoing person but this was eroded by her relationship with her ex-husband and she felt that lockdown had left her feeling very worried about crowded spaces or travelling. Her sons lived near the Midlands and she had missed seeing them. A holiday to Wales was planned but she felt very anxious about the drive. The team shared resources on anxiety management with her and encouraged engagement with her GP to manage her breathlessness symptoms and mental health. She was initially reluctant as felt that ‘*you never know who you’ll see there,’* but consented to a letter being sent from the team. She performed the repeated sit to stand test and achieved 2 reps in session two and three in the subsequent session. She cited back pain as a big limiting symptom for this exercise.

During session three she reported that her symptoms were gradually improving and her breathlessness was less evident during the video call. She had driven down to the holiday cottage in Wales and had a very pleasant time with her family there. She had enjoyed walks on the beach and the success of this holiday seemed to improve her confidence and self-efficacy. She had enjoyed a small amount of alcohol on holiday and was pleased that she had not craved an alcoholic drink since the holiday. She recalled *‘we had a big chat about my smoking, they want me to give up….and I will.’* At the end of the third session she said, ‘*I’m seeing things differently, talking to you two has helped me, soothed things a bit, calmed the waters.’* She wanted to engage with DWB after the end of the project. At the end of the project her EQ-5D-5L was 16 with improvements in self-reported anxiety and usual activities. Her FSS score was slightly improved at 59. She scored 5 on all domains of the PEM.

Participant eight

Participant eight was a 38-year-old BAME woman who lived with her two school age children. She was employed as a nurse and believed that she had caught COVID-19 at work

in March 2020. She had tested positive after becoming unwell at work with nausea, breathlessness and poor swallow. She briefly returned to work in May but felt unwell and was severely hypertensive. She continued to have symptoms of breathlessness and fatigue and returned to work in August 2020 on a shorter shift arrangement. At the start of the project she still experienced symptoms of breathlessness and fatigue. She was finding it hard to sustain her childcare commitments and the fathers of her children, from whom she was separated, only offered limited support. At the start of the project her EQ-5D-5L score was 9, with all domains mildly affected apart from self-care which was normal. Her FSS score was 33.

Before COVID-19 she recalled an active life. Although overweight and diagnosed with polycystic ovary syndrome, she used to take her children to the park and walk them to school. Since COVID she has felt too fatigued to take her children out and drives them to school. During session one, she became tearful, she described feeling tired but *'always having to get on, I can't really rest.'* Her job on the ENT ward demanded sustained physical activity and she was resistant to doing the sit to stand exercise as she described *'being on my feet all of the time.'* However, she was receptive to improving her physical wellbeing and wanted to lose weight, lower her blood pressure and prevent onset of diabetes. The team emphasised that it was important to be kind to herself alongside some changes to improve her physical health. She engaged with a stair walking programme combined with relaxation techniques.

During session three she reported improvements, she was walking part of the way to school and had taken the children to the park. The children had joined in with the relaxation activities which helped her to find time for this. She explained that she rarely opens up to people and felt a sense of relief for having talked about her mood and challenges she faced. She commented *'I'll remember you guys, thank you, thank you, for all you've done.'* At the end of the project her EQ-5D-5L score was 7 and her FSS 22. She scored 5 on all domains of the PEM.
